# Senescence and the Aging Immune System as Major Drivers of Chronic Kidney Disease

**DOI:** 10.3389/fcell.2020.564461

**Published:** 2020-10-09

**Authors:** Johannes Schroth, Christoph Thiemermann, Siân M. Henson

**Affiliations:** Translational Medicine and Therapeutics, William Harvey Research Institute, Barts and The London School of Medicine and Dentistry, Queen Mary University of London, London, United Kingdom

**Keywords:** aging, kidney, T cell, immune system, senescence

## Abstract

Chronic kidney disease (CKD) presents an ever-growing disease burden for the world’s aging population. It is characterized by numerous changes to the kidney, including a decrease in renal mass, renal fibrosis, and a diminished glomerular filtration rate. The premature aging phenotype observed in CKD is associated with cellular senescence, particularly of renal tubular epithelial cells (TECs), which contributes to chronic inflammation through the production of a proinflammatory senescence associated secretory phenotype (SASP). When coupled with changes in immune system composition and progressive immune dysfunction, the accumulation of senescent kidney cells acts as a driver for the progression of CKD. The targeting of senescent cells may well present an attractive therapeutic avenue for the treatment of CKD. We propose that the targeting of senescent cells either by direct inhibition of pro-survival pathways (senolytics) or through the inhibition of their proinflammatory secretory profile (senomorphics) together with immunomodulation to enhance immune system surveillance of senescent cells could be of benefit to patients with CKD.

## Introduction

Age-related pathologies are a major global disease burden, with potentially half of all morbidities being attributable to aging (Chang et al., 2019). Inflammation (or “inflammaging”) is one of the main causative factors contributing to disease progression, and has been described in various age-related pathologies, including type 2 diabetes (T2D) and cardiovascular disease (Franceschi et al., 2018). While being beneficial in the acute stages of an insult, inflammation increasingly fails to resolve with age, leading to changes in both cellular phenotypes and immune system composition. Senescence pathways are induced by, as well as potentiate, chronic inflammation, with increased cellular senescence being observed in various age-related diseases. Cellular senescence is characterized by a stable growth arrest and a proinflammatory secretome, which potentiates low grade chronic inflammation, thereby building a positive feedback loop, gradually exacerbating its effects on the body. With a prevalence of approximately 44% in the elderly population (>65 years), chronic kidney disease (CKD) presents a major disease burden in an aging population (Stevens et al., 2010). Therapies for late stage CKD including dialysis and renal transplantation carry a significant burden for patients, and the outcome is often poor (Roberti et al., 2018); therefore, there is a significant need for early diagnosis and novel therapies targeting mechanisms driving the disease. CKD is associated with chronic inflammation, elevated levels of cellular senescence, as well as immune system dysfunction. Their characterization as phenotypes or primary drivers of disease progression is crucial for the development of novel CKD therapies. The role of inflammation, senescence, and the immune system, with their potential modulation through therapeutics are discussed below.

## Chronic Kidney Disease—Incidence and Risk Factors

The human kidney undergoes various structural and functional changes with age. These include a decrease in renal mass, patterns of microstructural sclerosis (renal fibrosis), as well as changes in nephron number and a diminished glomerular filtration rate (Hommos et al., 2017). The global prevalence of CKD (at all stages) was estimated to be 9.1% in 2017 (Bikbov et al., 2020). Various risk factors, both inherited and environmental, have been identified to contribute to the development of CKD. Genetic risk factors include gene variants affecting creatine clearance (Bachmann et al., 2005; Köttgen et al., 2009), and glomerular filtration rates (Xu et al., 2018). Non-genetic risk factors such as smoking and nephrotoxins, as well as gender, ethnicity, and socioeconomic status increase the lifetime risk of developing CKD. Excessive comorbidity driven inflammation, for example, through type 2 diabetes (T2D) or obesity, is known to aggravate these processes, accelerating kidney dysfunction, leading to CKD and ultimately end stage renal disease (ESRD) (Liu et al., 2017). Diabetic kidney disease (DKD) is a major cause of ESRD and accounted for almost a third of disease adjusted life years of CKD in 2017 (Bikbov et al., 2020). Initiated by metabolic dysregulation, key contributors to DKD progression include the loss of tubular epithelial cells (TECs) and podocytes due to metabolic injury and ROS induced apoptosis (Susztak et al., 2006; Verzola et al., 2008). Frequent episodes of acute kidney injury (AKI) are also associated with progression to stage 5 CKD (ESRD) (Coca et al., 2012). AKI is defined by a sudden decrease in kidney function, often due to reduced renal blood flow and pre-existing health conditions, and is an independent risk factor of CKD incidence and progression (Coca et al., 2012). The mechanisms underlying the progression from AKI to CKD are not fully understood; however, production of fibrotic extracellular proteins mediated by TGF-β signaling in renal cells (Böttinger and Bitzer, 2002), as well as the p53 cell death signaling pathway has been described in tubular cells (Jiang et al., 2004). Crucially, proinflammatory/senescence and pro-apoptotic pathways have also been implicated in the development of CKD.

## Senescence and Inflammation in Premature Aging

Premature aging is a phenotype observed in many age-related pathologies such as T2D, rheumatoid arthritis, as well as in CKD (Franceschi and Campisi, 2014; Franceschi et al., 2018). Notably, individuals affected by these age-related diseases display similar phenotypes, including muscle wasting, vascular disease, osteoporosis, frailty, and immune dysfunction (Weiskopf et al., 2009; Crowson et al., 2010; Kooman et al., 2013; Schram et al., 2014). Given the similarity of these phenotypes, many of the underlying mechanisms are shared between them, in particular, genomic instability, changes in epigenetic modification, metabolic dysregulation, and cellular senescence (Kubben and Misteli, 2017). These changes are also observed in CKD, with patients exhibiting cellular alterations characteristic of increased inflammation and senescence. Additional causes of the premature aging observed in CKD include dialysis, interstitial sodium accumulation, uremia, as well as increases in angiotensin II and phosphate pools, which are expertly reviewed in Kooman et al. (2014, 2017).

### Cellular Senescence

Cellular senescence is a conserved mechanism by which cells exit the cell cycle in response to both intrinsic and extrinsic stresses. Two forms of senescence exist, replicative senescence and stress-induced premature senescence, discussed further below. Activation of senescence causes alterations in cell morphology, secretory phenotype, cell metabolism, and composition (Van Deursen, 2014). Senescent cells can be characterized by several defining markers, which have also been observed in CKD patients. Among these, the aging murine and human kidney express increased levels of senescence-associated-beta-galactosidase (SA-β-Gal), p16, and Ki-67 (Melk et al., 2005; Clements et al., 2013). Senescent cells also secrete a proinflammatory milieu termed the senescence associated secretory phenotype (SASP), implicating them in the progression of kidney dysfunction toward senescence. This has been shown in Wistar rats, where CKD progression induced multi-organ genomic damage and an increased expression of inflammatory markers (IL-1, IL-6, and TNFα) (Hirotsu et al., 2011). Senescence is further exacerbated by a reduction in anti-aging renoprotective factors and processes, including Klotho, mitophagy, vitamin D, and bone morphogenetic protein (Koh et al., 2001; Gould et al., 2002; Levin et al., 2007; Zhan et al., 2015). Klotho contributes to a cells ability to resist oxidative stress and reduces senescence when overexpressed in mice (Haruna et al., 2007). CKD patients produce significantly less Klotho (Koh et al., 2001), thereby accelerating the process of renal senescence. In addition, mitophagy is downregulated, particularly in renal tubular cells of DKD patients, resulting in dysfunctional mitochondria secondary to significant increase in mitochondrial ROS, which, in turn, drive stress-induced premature senescence (Zhan et al., 2015). Overexpression of the mitophagy inducing protein optineurin reduces cellular senescence in high glucose stimulated renal TECs (Chen et al., 2018). Accumulating senescent cells are normally cleared by the immune system. However, due to the age-associated decline in immune function, senescent cells remain in local tissues and contribute to tissue dysfunction and chronic inflammation. The underlying cellular mechanisms which lead to senescence in CKD are described further below.

### Sources of Senescence in CKD

Replicative senescence is caused by the progressive shortening of telomeres, which form the non-protein coding ends of human chromosomes. Telomeres shorten with every cell division, protecting protein-coding DNA from shortening, and can be elongated by the telomerase enzyme. At critically short lengths, telomeres reach their “Hayflick limit” (Hayflick and Moorhead, 1961), and recruit DNA damage repair machinery, which activates the p21 cyclin-dependent kinase inhibitor and causes cell cycle exit and senescence. In CKD, senescence is observed in a variety of cells, including TECs, podocytes, interstitial cells, and mesangial cells. The senescent cell type may vary between different types of CKDs. As such, the development of diabetic nephropathy is associated with the acceleration of replicative senescence in TECs, as well as an increased expression of p16 in podocytes and mesangial cells (Verzola et al., 2008). The involvement of the p21 pathway in CKD has been illustrated in a telomerase-deficient mouse model, which had a lower 30-day recovery rate after ischemia reperfusion injury (IRI) than controls (Westhoff et al., 2010). IRI, tissue damage caused by the reoxygenation of a tissue, is one of the most common causes of AKI and its frequency as well as duration determines the progression from AKI to CKD. Patients suffering from CKD routinely undergo hemodialysis treatment to replace renal function (renal replacement therapy). In mononuclear cells of hemodialysis patients, telomeres are shorter than in those from age-matched controls (Ramírez et al., 2005). These findings were replicated in renal transplant patients, showing that renal transplantation leads to greater telomere attrition than that observed in CKD patients undergoing dialysis (Luttropp et al., 2016). These findings show that the treatment of CKD with either hemodialysis or renal transplantation further contributes to peripheral cell senescence and, possibly to the dysfunction of the immune system. Alternatively, senescence can be induced in a premature manner, due to both intrinsic and extrinsic stressors (Table 1), including increased genomic damage, mitochondrial dysfunction, and oxidative stress, all of which are observed during CKD (Corredor et al., 2010; Gamboa et al., 2016).

**TABLE 1:** Inducers of cellular senescence.

Inducer	Mechanism	References
Telomere attrition	Critically short telomeres induce a DNA damage response which leads to the activation of senescence pathways	Hastings et al., 2004
Genotoxic agents/Irradiation	Irreparable DNA damage in response to genotoxic agents or irradiation	Yentrapalli et al., 2013
Oncogenes and tumor suppressor genes	Oncogene activation as well as suppression of tumor suppressor genes	Di Micco et al., 2006
Oxidative stress	Metabolic derived oxidative products induce DNA damage	Ziegler et al., 2015
Mitochondrial dysfunction	Decreased NAD+/NADH ratios drive senescence in an AMPK-dependant manner	Wiley et al., 2016
Epigenetic perturbations	Inhibition of DNA methylases and histone deacetylases induce senescence	Petrova et al., 2016
Paracrine mediators	SASP, particularly IL-1, induced senescence	Acosta et al., 2013

Senescence in the context of kidney function, however, is not all detrimental. Distinctions have to be made between acute and chronic senescence, as acute senescence has been observed to have beneficial effects. Anti-fibrotic mechanisms have been observed in murine models subjected to unilateral ureteral obstruction (UUO), which causes renal fibrosis due to tubular injury. INK4 knockout mice subjected to UUO reveal that p16^*INK4A*^ plays a pivotal role in limiting both inflammation and cell proliferation (Wolstein et al., 2010). Additionally, patients suffering from polycystic kidney disease have reduced expression of the senescence marker p21. In contrast, restoration of p21 expression in mice via cyclin-dependent kinase inhibition reduces disease progression (Bukanov et al., 2006). This beneficial effect of pro-senescence pathways has been recapitulated in p21 knockout mice subjected to renal IRI, exhibiting greater impairment in renal function as well as an increase in mortality when compared to their wild-type litter mates (Megyesi et al., 2001). These senescence pathways crucially halt the cell cycle and prevent the replication of damaged DNA. The sustained presence of senescent cells due to a failure to clear the senescent cell burden in the kidney leads to the continuous expression of profibrotic factors and a gradual deterioration of renal function. However, senescent cells may also accumulate owing to increased evasion strategies, which are discussed in subsequent sections.

### Inflammaging and the Senescence Associated Secretory Phenotype

The homeostatic regulation of inflammatory responses becomes aberrant in old age, favoring a chronic pro-inflammatory environment (Franceschi et al., 2006). The age-associated increase of a sterile, low-grade chronic inflammation is termed “inflammaging” and has been implicated in several age-related pathologies. Although normal aging is associated with an increase in chronic inflammation, various genetic and environmental factors may exacerbate inflammation and could explain the disparity between chronological and biological age in CKD patients (Kooman et al., 2014). Additionally, oxidative stress, acidosis, chronic infections, altered metabolism, and microbiome dysbiosis may also contribute to the inflammatory insult in patients with CKD. Together, these sources of inflammation compose the network theory of aging; where one component may lead to the exacerbation of another and thereby accelerates the overall aging process of the organism. Senescent cells secrete pro-inflammatory cytokines, the SASP, which is temporally dynamic and heterogeneous among cell types (Hernandez-Segura et al., 2017). Although the SASP can be beneficial by facilitating the recruitment of immune cells and promoting tissue damage repair, its inflammatory mediators also contribute to chronic inflammation, angiogenesis, and induce senescence of adjacent cells in a paracrine manner.

Chronic inflammation has long been implicated in CKD. Raj et al. analyzed plasma levels of pro-inflammatory mediators in 899 participants, finding that elevated levels of fibrinogen, TNFα, and IL-6 were associated with a more rapid progression of CKD (Amdur et al., 2016). Other studies have identified further inflammatory cytokines such as vascular endothelial growth factor (VEGF) and IL-10, as well as reporting a decreased renal clearance of these cytokines, likely due to uremia-induced lymphocyte dysfunction. Urea also induces endothelial progenitor cell senescence, contributing to the SASP in a ROS-dependent manner (D’Apolito et al., 2017). Upon kidney injury, factors such as VEGF and fibroblast growth factor-2 (FGF2) facilitate tissue remodeling, while pro-inflammatory cytokines facilitate the recruitment of immune cells. When the immune cell clearance of apoptotic or damaged cells fails, these cytokines are expressed chronically and contribute to the involvement of the SASP in the age-related pathological damage observed in CKD patients (Wang et al., 2017). Together, these findings describe the multifactorial nature of inflammation in CKD. Dysregulation of signaling-, metabolic-, and inflammatory-pathways, as well as decreased proliferation are features of senescent cells in CKD.

## Immune System Alterations

**FIGURE 1:**
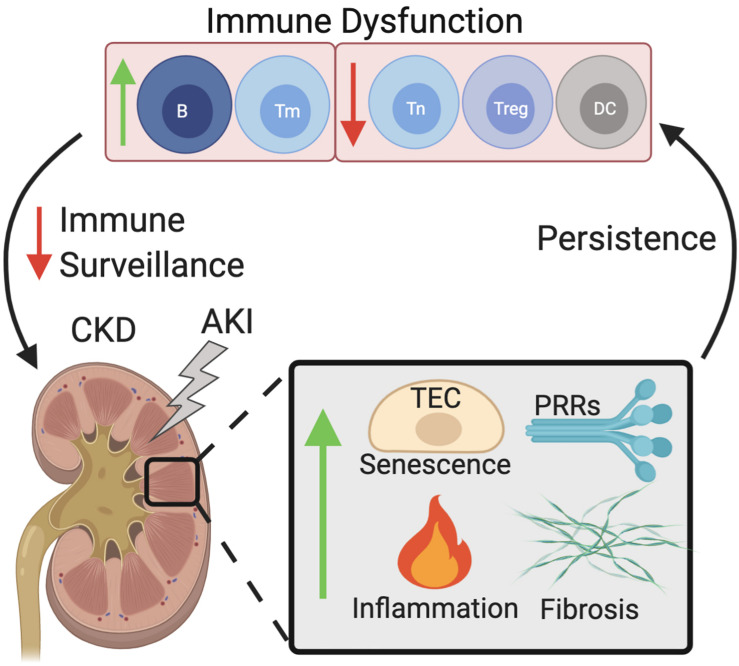
Dysfunctional immune senescence caused by persistent senescence, inflammation, and fibrosis of the kidney. AKI increases senescence of TECs, kidney fibrosis, inflammation, as well as increased PRR expression. Persistence of these effects leads to immune dysfunction which in turn decreases immune surveillance of the kidneys, exacerbating the acute effects of AKI which then manifest as CKD. Abbreviations: AKI, acute kidney injury; TEC, tubular epithelial cell; PRRs, pattern recognition receptors; DC, dendritic cell; B, B cell; Treg, regulatory T cell; Tm, memory T cell; Tn, Naïve T cell; CKD, chronic kidney disease.

Both the innate and adaptive immune systems are implicated in CKD progression (Figure 1). Senescence of tubular cells is particularly driven by the innate immune system, where AKI causes the infiltration of innate immune cells via Toll-like receptors (TLRs), and IL-1R signaling promotes senescence, mainly of TECs (Jin et al., 2019). In patients suffering from chronic renal failure, sera levels of pattern recognition receptors are also dysregulated, with increased mannose binding lectin levels (Atsushi et al., 2002), and increases in the macrophage scavenger receptors (Ando et al., 1996; Chmielewski et al., 2005). Monocytes and monocyte-derived dendritic cells (moDC) cultured in high concentrations of urea show decreased levels of endocytosis and poor maturation (Lim et al., 2007) and the terminal differentiation of moDCs is impaired in CKD patients, resulting in impaired antigen presentation and a decreased production of antigen-specific T cells (Verkade et al., 2007). FGF is elevated in CKD and contributes to impaired leukocyte recruitment to inflamed tissues via interference of leukocyte-integrin activation (Rossaint et al., 2016). These signaling pathways also induce a local SASP and are thereby implicated in aiding the progression of AKI toward CKD. As such, the senescence driven by CKD is mainly affected by local tissue changes, while senescent cell effector molecules (the SASP) contribute to both local and systemic changes in immune dysfunction. Additionally, CKD associated conditions such as uremia further potentiate this response by dysregulating immune cell function.

The adaptive immune response is also affected in CKD, with an increased presence of CD4+CD28− highly differentiated T cells, a reduced number of Tregs, and an overall decrease in proliferation rates all being observed (Lisowska et al., 2012). Patients in ESRD also have a reduced CD4:CD8 T cell ratio, suggestive of poor outcomes, as well as a selective depletion of naïve and central memory T cells (Yoon et al., 2006). A progressive decrease in renal function is associated with a selective loss of naïve and memory CD4 T cells, as well as an increase in CD8 memory T cells, which lack CD45RO and CCR7. A shift toward the pro-inflammatory Th1 differentiation is also observed (Litjens et al., 2006). In addition to the cellular immune response, humoral immunity is also impaired in CKD patients. Hemodialysis patients exhibit peripheral B cell lymphopenia (Fernández-Fresnedo et al., 2000), while a decrease in immature B cells has been reported in pre-dialysis ESRD patients (Kim et al., 2012). Notably, this immature B cell population inhibits the differentiation of proinflammatory CD4 T cells (Blair et al., 2010), illustrating the interlinked nature of cellular and humoral immune response dysregulation in CKD patients. Thereby the defective immune system leads to the accumulation of senescent cells in inflamed tissues that cannot efficiently be cleared.

The accumulation of senescent cells with age is not only due to reduced immune surveillance but also immune evasion. Perforin knockout mice which exhibit an impairment in immune cytotoxicity accumulate larger amounts of senescent cells (in all tissues) than their wild-type litter mates, which is accompanied by chronic inflammation and tissue fibrosis (Ovadya et al., 2018). Senescent cells, much like cancer cells, express receptors which activate inhibitory immune cell receptors, thereby evading immune cell clearance. Senescent fibroblasts accumulate the non-classical MHC receptor HLA-E in response to SASP signaling, which interacts with the inhibitory NK receptor, NKG2A expressed by NK cells, and highly differentiated CD8 T cells (Pereira et al., 2019). Both immune dysfunction and immune evasion contribute to the accumulation of senescent cells; however, discerning between the driver and product of CKD is crucial for identifying therapies.

## The Chicken or the Egg

Senescent cells may both be a phenotype of age-related inflammatory disease, as well as the cause for disease progression. Thereby two models of disease progression exist: One in which senescent cells arise from local tissue injury, promoting senescence in neighboring cells in a paracrine manner. Alternatively, immune clearance may be impaired, thereby allowing the accumulation of senescent cells. Distinguishing between these two models becomes pivotal when exploring potential new treatments of CKD.

Various renal diseases including AKI, diabetic nephropathy, and glomerulonephritis may lead to CKD. AKI is the main driver of CKD, where a rapid deterioration in kidney function often results in incomplete tissue repair. As described above, senescence is protective in response to AKI, with its SASP facilitating immune cell clearance and tissue repair. However, failure to clear these cells may lead to chronic SASP signaling and CKD. The importance and often bifunctional role of the immune system in AKI to CKD progression have been reported extensively. This includes proinflammatory infiltrating T and B cells which in response to tissue injury, contribute to a pro-fibrotic milieu, activating pericytes and inducing renal fibrosis and thereby CKD (Lee et al., 2017). Alternatively, CD4 and CD8 T cell depletion studies have illustrated their reno-protective effects in an acute aristolochic acid nephropathy model (Baudoux et al., 2018). Anti-inflammatory immune cells generally prevent the progression to CKD. M2 macrophages promote epithelial tissue repair process following IRI (Lee et al., 2011), as well as regulatory T cells promoting kidney recovery following AKI (Kinsey et al., 2013). The transcription of proinflammatory cytokines is largely regulated by the NF-kB transcription factor. Johnson et al. (2017) have reported that inhibition of the activation of NF-kB reduces renal fibrosis after AKI.

Much of the immune dysregulation is governed by the presence of proinflammatory cytokines, and uremia, or by pre-existing comorbidities such as high blood pressure or diabetes. In addition to the levels of inflammatory markers, an elevated white blood cell count is predictive of CKD development (Shankar et al., 2011). This suggests various modes of pathogenesis, with the immune system, senescence, and inflammation at its core. Cellular senescence and immunosurveillance occur in conjunction, increased cellular senescence due to chronic inflammation increases the demand for immune clearance; however, as described above, uremia and inflammation lead to dysfunction of the immune system, thereby establishing a positive feedback loop in which more senescent cells drive inflammation and immune dysregulation which, in turn, cause more senescence.

## Therapeutic Targeting of Senescent Cells in CKD

**FIGURE 2:**
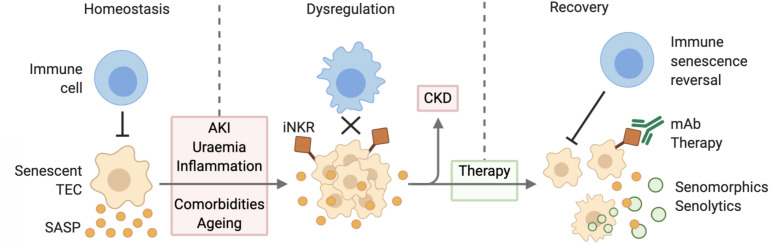
Senescent cell accumulation due to defective immune surveillance leading to CKD. During homeostatic conditions, immune cells target senescent TECs and are thereby able to mitigate SASP expression. AKI, uremia, inflammation, comorbidities, and aging lead to the dysregulation of this mechanism causing senescent cell accumulation and increased SASP expression. Senescent TEC expression of inhibitory receptors further decreases their clearance. Over time this may progress to CKD. Therapy via senolytic and senomorphic therapies, as well as immunomodulation improve senescent cell clearance and thereby contribute to reduced inflammation and recovery. Abbreviations: TEC, tubular epithelial cell; SASP, senescence associated secretory phenotype; AKI, acute kidney injury; iNKR, inhibitory natural killer cell receptor; CKD, chronic kidney disease; mAb, monoclonal antibody.

There has been a growing appreciation of the therapeutic potential of eliminating senescent cells to treat or prevent the onset of age-related disease. The first proof of this concept came with the engineering of a transgenic mouse with a drug inducible transgene of p16^*INK4a*^, showing that elimination of p16^*INK4a*^ positive senescent cells delayed the onset of age-related pathologies in tissues particularly prone to senescence (Baker et al., 2011). Inhibition of the SASP may also have reno-protective effects, dampening local tissue inflammation and preventing the paracrine signaling mediated induction of senescence in neighboring cells. And finally, the treatment of age-related immune dysfunction may be the most effective strategy, as it would activate the body’s natural targeting of senescent cells, promoting tissue repair and dampening inflammation (Figure 2).

### Senomorphics

Drugs which inhibit SASP inducing pathways (senomorphics) alleviate the aging phenotype of various age-related diseases (Table 2). Clinical trials have begun to show efficacy of such interventions in CKD. Among these, sodium-glucose cotransporter 2 inhibitors affect renal hemodynamics and have reno-protective effects (Yaribeygi et al., 2018). In addition, p38 MAPK inhibitors reduce SASP cytokines in a variety of cell lines (Alimbetov et al., 2016) including kidney glomerular and mesangial cells (Stambe et al., 2003; Wang et al., 2013), while oral administration of the p38 MAPK inhibitor SB203580 improves renal function and decreases proteinuria in a systemic lupus erythematosus (MRL/lpr) mouse model (Jin et al., 2011). Resveratrol, an anti-inflammatory and antioxidant polyphenol, inhibits renal fibrosis via the activation of *SIRT1* and deacetylation of *SMAD3* in UUO mice (Li et al., 2010), and decreases the renal fibrosis caused by diabetic hyperglycemia activated renal fibroblasts via the inhibition of AMPK/NOX4/ROS signaling (He et al., 2016). However, clinical trials in CKD patients have thus far failed to replicate these beneficial preclinical effects (Saldanha et al., 2016). The SASP activating transcription factor NFκB is a primary target for inhibition in CKD. In a rat model receiving adenine overload, administration of the IκB kinase inhibiting compound pyrrolidine dithiocarbonate markedly reduces macrophage infiltration and attenuates renal interstitial fibrosis (Okabe et al., 2013). The naturally occurring NFκB inhibitor parthenolide ameliorates renal injury and inflammation in cisplatin-induced renal damage models (Francescato et al., 2007), as well as reducing renal monocyte and macrophage infiltration in UUO models (Esteban et al., 2004). Its antifibrotic properties have shown efficacy in patients suffering from diabetic nephropathy and glomerulosclerosis (Cho et al., 2007; Sharma et al., 2011). In addition, inhibition of the activation of NF-kB with an inhibitor of IKK after AKI (peak in creatinine) prevents the subsequent development of renal fibrosis, a key driver of the development of CKD (Johnson et al., 2017).

**TABLE 2:** Senomorphics.

Inhibitor(s)	Function	References
SGLT2 inhibitors: Empagliflozin Dapagliflozin Canagliflozin	Affect renal hemodynamics and inhibition of proinflammatory cytokine production	Yaribeygi et al., 2018
BIRB796 UR-13756 SB203580	p38 MAPK inhibition	Alimbetov et al., 2016
Resveratrol	Pleiotropic effects, including *SIRT1/SMAD3* inhibition	Li et al., 2010
Pyrrolidine dithiocarbonate Parthenolide	NFκB inhibitors	Francescato et al., 2007; Okabe et al., 2013

While senomorphics have shown to be potent inhibitors of the SASP, several challenges remain. Primarily, the clearance of SASP inhibited senescent cells proves difficult, as immune cells may fail to recognize these for clearance. *In vitro* senomorphic treatment of senescent fibroblasts has resulted in the downregulation of SASP mediators involved in immune cell recruitment, such as CXCL1 and GM-CSF (Lim et al., 2015). Additionally, treatment would require chronic administration of senomorphic agents, which would likely result in undesirable side effects due to their non-specific targeting of senescent cells. A brief and efficacious treatment course would thereby have to be achieved.

### Senolytics

Pharmacological agents targeting characteristic cellular mechanisms and molecular features of senescent cells have been termed “senolytics.” Identification of senescent-cell anti-apoptotic pathways (SCAPs) has allowed the development of specifically targeted senolytics for each of these. Additionally, senescent cells are highly metabolically active, and metabolically constrained, thereby providing further targeting phenotypes (Zhu et al., 2015). Several murine studies have shown that the clearance of senescent glial cells prevents cognitive decline (Bussian et al., 2018), while the elimination of p19^*ARF*^ expressing cells enhances pulmonary lung function in 12-month-old mice (Hashimoto et al., 2016). Pre-clinical studies have identified a variety of SCAP interfering molecules, such as various BCL family inhibitors, ABT-263 (navitoclax) (Chang et al., 2016), ABT-737 (Yosef et al., 2016), and fisetin (Pal et al., 2013; Zhu et al., 2017), as well as dasatinib and quercetin which have a wide variety of pro-apoptotic and anti-survival effects (Zhu et al., 2015) (Table 3). In patients suffering from DKD, coadministration of dasatinib and quercetin reduces senescent cell burden in adipose and skin epidermal tissues, as well as circulating SASP factors (Hickson et al., 2019). Additionally, inhibition of the FOXO4-p53 interaction, which mediates senescent cell specific p53 nuclear exclusion and hence, apoptosis, restores fitness and renal function in naturally aged mice (Baar et al., 2017).

**TABLE 3:** Senolytics.

Inhibitor(s)	Function	References
Dasatinib and quercetin	Bcl-2, PI3K, TK inhibitors	Zhu et al., 2015; Hickson et al., 2019
Fisetin	Bcl-2 family inhibitor	Zhu et al., 2017
Navitoclax (ABT-263)	Bcl-2 family inhibitor	Chang et al., 2016
ABT-737	Bcl-2 family inhibitor	Yosef et al., 2016
FOXO4-DRI	FOXO4-p53 interaction inhibitor	Baar et al., 2017

Similar to senomorphic treatments, the senolytic targeting of senescent cells has resulted in undesirable off target effects. As shown in mouse models, this may be overcome by using nanocapsules, which target senescence specific proteins, thereby only releasing the senolytic upon senescent cell contact (Muñoz-Espín et al., 2018). Additionally, it is unclear whether apoptotic senescent cells can be efficiently cleared by a dysfunctional immune system. While senolytics may improve immune function by targeting senescent immune cells, immune mediated CKD may be exacerbated further via increased immune activation and inflammation. Thereby it is likely that senolytic treatment needs to be occur in conjunction with immunomodulatory treatments to achieve the specific targeting and clearance of senescent cells in the kidney.

### Immunomodulation

Given the assumption that defective immune mediated senescent cell clearance is at the root of senescent cell accumulation and, thereby, its proinflammatory environment, modulation of the immune system would be the most effective treatment in age-related diseases. This modulation may come in various categories, including reversal of the proinflammatory senescent state of immune cells, induction of tolerance in response to acute injury, as well as improving senescent cell clearance by modulation of homing and targeting capabilities. Inhibition of p38 MAPK in senescent CD8 T cells, for example, increases their proliferation, telomerase activity, and mitochondrial biogenesis (Henson et al., 2014). Peripheral tolerance is largely controlled by DCs via the induction of Treg cells as well as T cell anergy. Induction of tolerogenic DC populations, via *ex vivo* adenosine 2A receptor agonist treatment, has reno-protective effects in experimental IRI by suppressing NKT cell activation (Li et al., 2012). Various immunotherapies may be applicable in the clearance of senescent cells, such as vaccines, reinfusion of *ex vivo* derived DCs, and chimeric antigen receptor (CAR) T cells. In a proof of concept study, Truong et al. engineered a chimeric IL-6 receptor which under IL-6 stimulation generated a Ca^2+^ signal. This was co-expressed with a Ca^2+^ activated RhoA, enabling migration of cells toward IL-6 and subsequent fusion of cells, leading to targeted cell death (Qudrat et al., 2017). Additionally, improvement of immune clearance of senescent cells can be achieved by blocking the inhibitory HLA-E:NKG2A signaling axis between senescent fibroblasts and NK cells and late differentiated CD8 T cells (Pereira et al., 2019).

Immunomodulation is likely a key therapeutic method to target senescent cells in CKD. Mitigation of age-related immune dysfunction in conjunction with improved targeting of senescent cells ensures a non-exaggerated immune response as well as the elimination of senescent cells. However, this strategy relies on the presence of conserved senescence specific markers. Tissue specific evaluation of senescent cell markers is therefore required.

## Conclusion

Chronic kidney disease presents an ever-increasing global health burden. Its association with age and chronic inflammation identifies senescent cells as the main culprit of the deterioration of kidney structure and function. Both changes in the immune system as well as the accumulation of senescent cells within the kidney are associated with the progression of kidney disease. Targeting of both aspects will be necessary to preserve long-term beneficial effects of therapy. However, with advancing insights come major challenges, which will need to be tackled before these therapies will show efficacy in patients. The pleiotropic effects of senomorphics will need to be studied extensively to avoid unwanted effects. Optimization of dosing and the limitation of adverse effects still present significant challenges. Finally, the involvement of the immune system in the progression of CKD due to immune-senescence and immune-evasion need to be studied extensively to achieve the ultimate trifecta of therapy, by eliminating senescent cells, blocking their SASP signal, and promoting immune cell targeting of senescent cells.

## Author Contributions

JS wrote the manuscript. CT and SH proofed and advised on the content. All authors contributed to the article and approved the submitted version.

## Conflict of Interest

The authors declare that the research was conducted in the absence of any commercial or financial relationships that could be construed as a potential conflict of interest.
